# Susceptibility of consolidated procedural memory to interference is independent of its active task-based retrieval

**DOI:** 10.1371/journal.pone.0210876

**Published:** 2019-01-17

**Authors:** Ella Gabitov, Arnaud Boutin, Basile Pinsard, Nitzan Censor, Stuart M. Fogel, Geneviève Albouy, Bradley R. King, Julie Carrier, Leonardo G. Cohen, Avi Karni, Julien Doyon

**Affiliations:** 1 Functional Neuroimaging Unit, C.R.I.U.G.M., Montreal, Quebec, Canada; 2 McConnell Brain Imaging Center, Montreal Neurological Institute, Montreal, Quebec, Canada; 3 Sorbonne Université, CNRS, INSERM, Laboratoire d’Imagerie Biomédicale, LIB, Paris, France; 4 School of Psychological Sciences and Sagol School of Neuroscience, Tel Aviv University, Tel Aviv, Israel; 5 School of Psychology, University of Ottawa, Ottawa, Ontario, Canada; 6 Movement Control and Neuroplasticity Research Group, Department of Movement Sciences, KU Leuven, Leuven, Belgium; 7 Research Center of Sacré-Cœur Hospital of Montreal, Montreal, Quebec, Canada; 8 Human Cortical Physiology and Neurorehabilitation Section, National Institute of Neurological Disorders and Stroke, National Institutes of Health, Bethesda, Maryland, United States of America; 9 Laboratory for Human Brain & Learning, Sagol Department of Neurobiology & the E.J. Safra Brain Research Center, University of Haifa, Haifa, Israel; University of California San Diego, UNITED STATES

## Abstract

Reconsolidation theory posits that upon retrieval, consolidated memories are destabilized and need to be restabilized in order to persist. It has been suggested that experience with a competitive task immediately after memory retrieval may interrupt these restabilization processes leading to memory loss. Indeed, using a motor sequence learning paradigm, we have recently shown that, in humans, interference training immediately after active task-based retrieval of the consolidated motor sequence knowledge may negatively affect its performance levels. Assessing changes in tapping pattern before and after interference training, we also demonstrated that this performance deficit more likely indicates a genuine memory loss rather than an initial failure of memory retrieval. Here, applying a similar approach, we tested the necessity of the hypothetical retrieval-induced destabilization of motor memory to allow its impairment. The impact of memory retrieval on performance of a new motor sequence knowledge acquired during the interference training was also evaluated. Similar to the immediate post-retrieval interference, interference training alone without the preceding active task-based memory retrieval was also associated with impairment of the pre-established motor sequence memory. Performance levels of the sequence trained during the interference training, on the other hand, were impaired only if this training was given immediately after memory retrieval. Noteworthy, an 8-hour interval between memory retrieval and interference allowed to express intact performance levels for both sequences. The current results suggest that susceptibility of the consolidated motor memory to behavioral interference is independent of its active task-based retrieval. Differential effects of memory retrieval on performance levels of the new motor sequence encoded during the interference training further suggests that memory retrieval may influence the way new information is stored by facilitating its integration within the retrieved memory trace. Thus, impairment of the pre-established motor memory may reflect interference from a competing memory trace rather than involve interruption of reconsolidation.

## Introduction

The traditional consolidation hypothesis posits that new memories are initially labile and susceptible to interference, but become stable in the long term through a protein-synthesis-dependent process known as “consolidation” [1–3]. In the late 1960s, this view, however, was challenged due to evidence that, in rats, electroconvulsive shock induced loss of previously consolidated memory, but only if animals were exposed to a “reminder cue” [4]. Over the last two decades, similar loss of consolidated memory has been repeatedly demonstrated using, initially, pharmacological and, lately, behavioral interference in close temporal proximity to memory retrieval [5–15], but see [16]. Thus upon retrieval, consolidated memories are reactivated and may return to a labile state becoming, once again, susceptible to interference. Crucially, interference procedures after memory retrieval have been shown to be effective only if they were administered within a limited time-window, that is when memories were still “active”. These findings were interpreted as support to the reconsolidation hypothesis, which postulates that memory retrieval can lead to memory destabilization, thereby necessitating another consolidation-like period of protein-synthesis-dependent stabilization, called “reconsolidation” [17]. Extending the reconsolidation explanation to memory enhancement, it has been proposed that the post-retrieval restabilization time-window constitutes a unique opportunity for potential adaptive memory modification [18]. Importantly, the underlying mechanisms of memory strength modification implied by reconsolidation are separate from those of memory acquisition and consolidation [19,20].

The reconsolidation theory considers retrieval as a necessary condition for mechanistic instantiation of memory updating process, interruption of which may lead to memory loss [5,6,9,19]. However, some animal studies suggest otherwise, hence providing evidence for the loss of consolidated memory upon pharmacological inhibition of retrieval [21–24]. The primary aim of the present study was thus to test whether active task-based memory retrieval is necessary to induce impairment of consolidated memories by interference experience in humans. Since pharmacological interventions are problematic and are often associated with increased risk in human subjects, we used a competing task as a post-retrieval interference manipulation. This approach is often considered as a safe alternative to injections of protein synthesis inhibitors to study susceptibility of consolidated memories to interference in humans [9,10,14,15]. Similar to pharmacological protocols, susceptibility of consolidated memory to behavioral interference is usually tested using a 3-day study design. On the first experimental day, a new memory is encoded during experience with a particular stimulus or task. On the second experimental day, this memory is then retrieved (or not) and manipulated by providing an interference experience with a new, competing stimulus or task. Finally, on the last experimental day, the strength of the memory is tested.

Recently, using a computerized version of the sequential finger tapping task adapted from Karni et al. (1995, 1998), we tested time-dependent effects of post-retrieval behavioral interference on a consolidated motor skill, a form of procedural (“how to”), practice-dependent memory, in humans [25]. In that study, we optimized the parameters of a 3-day motor sequence learning paradigm [9,10,16] and showed that only immediate post-retrieval interference, but not after an 8-hour delay, resulted in performance deficit of a consolidated motor skill on the following day. During the experiment, participants practiced one sequence on Day 1 (T-Seq), briefly performed that sequence (memory retrieval) and then practiced another new sequence (i.e., interference training; Int-Seq) on Day 2 and, finally, took part in a test session of both sequences on Day 3. The interference training was given either immediately or 8 hours after memory retrieval (ReInt and Re8hInt group, respectively). The impaired performance of the initially trained sequence in the ReInt group was expressed as a loss of post-training delayed gains in performance, a behavioral manifestation of motor sequence memory consolidation [9,26–28]. These gains were preserved in the Re8hInt group, similar to a control group that on Day 2 underwent memory retrieval without subsequent interference training. In line with previous studies [10,29,30], the detrimental effects of immediate post-retrieval interference were transient and performance levels in the ReInt group rapidly recovered with continued practice, raising the possibility of an initial failure in memory retrieval rather than its genuine decay [31]. This issue was addressed by estimating experience-related changes in motor serial task representations that are presumably reflected in an actual tapping pattern of the sequence [32,33]. We showed that performance improvement during the final test on Day 3 in the ReInt group was paralleled by progressive formation of a new tapping pattern and, therefore, most likely indicated new learning. No evidence for recovery of previously formed tapping patterns was found. Thus, the explanation of rapid rescue of previously established motor sequence knowledge following initial failure in its retrieval was not supported.

**Fig 1:**
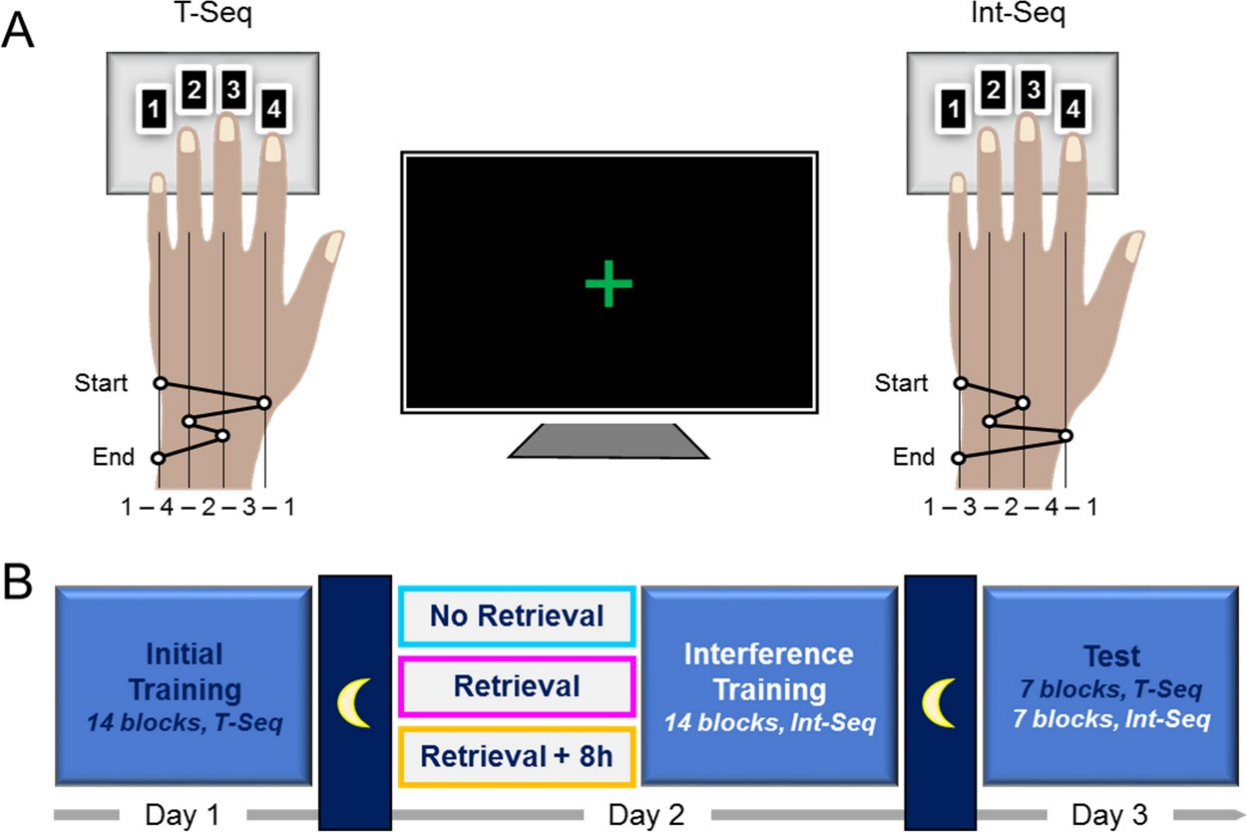
Study design. (A) sequential finger tapping task. A sequence initially trained on the Day 1 (T-Seq, left panel) and a novel sequence used during the interference training on Day 2 (Int-Seq, right panel). The two sequences were matched for number of movements per digit and mirror-reversed in relation to each other (in terms of order). (B) Experimental groups. On Day 1 all groups underwent training on the T-Seq consisted of 14 performance blocks. On Day 2 the memory for the T-Seq was retrieved (or not) using one performance block and the interference training on the novel Int-Seq was conducted according to the experimental group (NoReInt, ReInt and Re8hInt). On Day 3 the performance levels for the T-Seq were tested in all groups using 7 performance blocks; the performance for the Int-Seq was subsequently tested as well. In all sessions performance blocks consisted of 60 key-presses, equivalent to 12 possible sequences, and were separated by 25-second periods of rest.

Here, to examine the necessity of memory retrieval to induce deficit in the pre-established motor skill, we tested an additional group using similar approach as described above but without active task-based retrieval of the T-Seq before interference training (NoReInt group) (Fig 1). Statistical inferences were made by comparing this group with the ReInt group. The Re8hInt group was used as a control group. We allowed for the possibility that without any additional experience with the T-Seq on Day 2, participants may have initial difficulty in memory retrieval and may show impaired performance on the following day regardless of the effectiveness of interference training. Based on our previous results [25], we predicted that in that case, the tapping pattern of the T-Seq during the test on Day 3 would become progressively more similar to the tapping pattern formed by the end of the initial training, indicating successful memory retrieval with continued practice. However, impaired performance of the T-Seq on Day 3 followed by formation of a new tapping pattern would indicate that previously-formed representations of the T-Seq are not available/ not accessible and that a new memory trace need to be rooted.

Whereas studies on reconsolidation highlighted that consolidated memories are destabilised upon their retrieval, other evidence suggest that an effective retrieval experience can influence the way new information is stored [1,34,35]. Therefore, we also tested the impact of memory retrieval on acquisition of a new motor sequence knowledge encoded during the interference training by analysing data of the Int-Seq.

## Methods

### Ethics statement

All participants gave their written informed consent to take part in the study, which was approved by the Research ethics board of the RNQ (Regroupement Neuroimagerie Québec). All procedures were in accordance with the approved guidelines and regulations. Subjects were compensated for their participation.

### Participants

For the current report we extended our previous work [25] by including an additional group. The current report is based on the analyses of data from 50 healthy young right-handed [36] adults (mean age = 24.42, SD = 3.75, 28 females) including data from 35 participants (ReInt and Re8hInt group) recruited previously. Participation in the study required that subjects are able to perform and learn the motor task. All participants reported no prior history of neurological or psychiatric illness, no brain injury and no addiction to drugs, alcohol or cigarettes (i.e., subjects were non-smokers or occasional smokers). Exclusion criteria included the current or chronic use of medication, any known learning disabilities and an attention deficit disorder. Only individuals with less than 1 year of formal music training participated in the current study. Professional typists and experienced gamers were excluded as well. All participants had normal quality of sleep, as assessed by the Pittsburgh Sleep Quality Index questionnaire [37], and reported at least 6 hours of proper nocturnal sleep night before each experimental session. For follow-up, participants were asked to fill the Consensus Sleep Diary [38] (S1 Table). Throughout the experiment, participants were asked to refrain from drinking alcohol, not to take naps and to follow their normal sleep schedule. Between experimental sessions, they were instructed to continue with their regular routine.

### Motor sequence learning task

The task protocol used in the current study has been described in detail elsewhere [25]. Briefly, the participants were required to perform a computerized version of the sequential finger tapping task adapted from Karni et al. (1995, 1998) implemented using the Psychophysics Toolbox extensions for Matlab (The Mathworks, Inc., Natick, MA). Lying supine, participants were instructed to tap “as fast and accurate as possible” a 5-element sequence of finger movements with their left (non-dominant) hand using a 4-key response pad (Fig 1A). During each session, periods of rest (25 s) and performance (i.e., performance blocks, 60 key-presses) were marked by visual stimuli (red and green fixation cross, respectively) presented in the middle of the screen. This protocol controlled for the number of movements executed per block to ensure that the same sensory-motor experience with the task was afforded across participants during a particular session. Throughout the experiment, no feedback was afforded.

### Design and experimental procedure

The study consisted of three experimental phases, each taking place on three consecutive days: (1) initial training, (2) interference training with / without prior retrieval of the consolidated memory and (3) testing (Fig 1B). For each participant, each experimental phase started at approximately the same time of day to minimize putative impact of circadian and homeostatic factors on individual performance levels throughout the experiment [39]. During training, participants were instructed to perform a sequence, 1-4-2-3-1 (T-Seq) on Day 1 and 1-3-2-4-1 (Int-Seq) on Day 2, for 14 successive performance blocks. On Day 2, each participant was assigned to one of the following conditions: interference training without memory retrieval experience, memory retrieval juxtaposed to interference training and memory retrieval separated from interference training by 8-hour delay (NoReInt group, n = 15; ReInt group, n = 20; Re8hInt group, n = 15, respectively). In the ReInt and Re8hInt group, memory for the T-Seq was retrieved using a single performance block. Participants in the Re8hInt group left the laboratory after the retrieval experience. They were instructed to continue with their regular routine and were re-exposed to the experimental environment during the interference training 8 hours later. Thus, participants of each group took part in two equivalent training sessions practicing the T-Seq on Day 1 and the Int-Seq on Day 2. The only difference between the groups was the existence and timing of the short re-exposure to the T-Seq through its actual performance prior to the second interference training session. During the test on Day 3, all participants were instructed to perform the T-Seq, followed by the Int-Seq, tapping each sequence for 7 successive blocks. The decision to test the T-Seq first rather than using a counterbalanced design was made to exclude the possibility of proactive interference from additional experience with the Int-Seq and task-switching cost on the T-Seq performance during the test [40]. In this way, we could disentangle the impact of memory retrieval on the effectiveness of behavioral interference to induce deficit in a pre-established motor skill by comparing changes in performance over the post-training interval for the T-Seq in subjects with similar experience with the task. Moreover, the test of the Int-Seq on Day 3 allowed us to estimate how retrieval experience influences the way a new motor sequence knowledge is encoded and consolidated. Although we could not rule out the possibility of proactive interference caused by additional experience with the T-Seq prior to the test of the Int-Seq, given the same order in all groups we assumed that greater post-training gains for the Int-Seq will reliably reflect stronger memory.

### Data analyses

Performance measures were calculated for each participant and, unless otherwise stated, the analyses were designed as mixed repeated measures Analysis of Variance (ANOVA) using individual values as a within-subjects factor and group as a between-subject factor. The results were corrected for non-sphericity violation using the Greenhouse-Geisser adjustment, when appropriate.

It has been consistently shown that experience and memory processes for explicitly known motor sequences are associated with substantial changes in performance rate while the number of errors is extremely low [27,28,41,42] (for accuracy rates observed in the current study, please, consult S1 File). Therefore, in the current study, the end-point measure for motor sequence task performance was a measure reflecting performance rate, i.e., the time (duration) per block spent at performing the motor sequence task [41,43]. To minimize the effects of additional practice during test sessions, skill levels were evaluated using data from a single block only. Furthermore, to account for warm-up effects and an underestimation of the actual level of skill acquired [44], performance measures were assessed using data from the final 30 key-presses (i.e., equivalent to 6 final sequences of the block; see also [45]). Post-training gains in performance rate were determined for each sequence using a percentage score based upon performance levels achieved during the last block of training so that positive values correspond to faster performance by Day 3 than by the end of training.

Changes in motor sequence representations were assessed by testing for experience-driven changes in a tapping pattern of a sequence. To this end, inter-key press intervals (IPIs), i.e., durations between successive key presses, of all correctly completed sequences were extracted for each performance block of interest, being classified according to one of 4 possible transitions between successive elements within a sequence plus an additional transition between the sequences. Average IPIs were then calculated separately for each of the five possible transitions excluding any value that was above or beyond two standard deviations from the mean. This procedure resulted in 5-element vectors of IPIs that represent individual tapping patterns of a sequence implemented during performance at different time-points of interest. Experience-driven changes in tapping patterns were assessed using normalized Pearson correlation coefficients calculated between two tapping patterns at different time-points of interest. The normalization was performed using Fisher z-transformation. Note that the correlation coefficients indicate the degree of similarity between tapping patterns (i.e., higher values corresponding to greater pattern similarity, and vice versa), but do not reflect changes in overall performance rate. Moreover, this approach takes into account the inter-subject differences in the way the tapping pattern is formed and modified by previous experience with the task [32,46,47], and allows to perform statistical inferences without making any assumption about the shape and experience-driven changes of the tapping pattern at the group level [25].

## Results

### Encoding of a new motor sequence knowledge

The susceptibility of the pre-established memory to interference may depend on the efficiency of the initial training and memory trace dominance [48]. Therefore, through analysis of the data acquired during training sessions, we first validated that motor skill levels during memory encoding were comparable across groups. The equivalent experience afforded during the training of each sequence also allowed us to test for the impact of previous experience with the task (T-Seq) on acquisition of a new motor sequence knowledge (Int-Seq).

#### Performance rate

**Fig 2:**
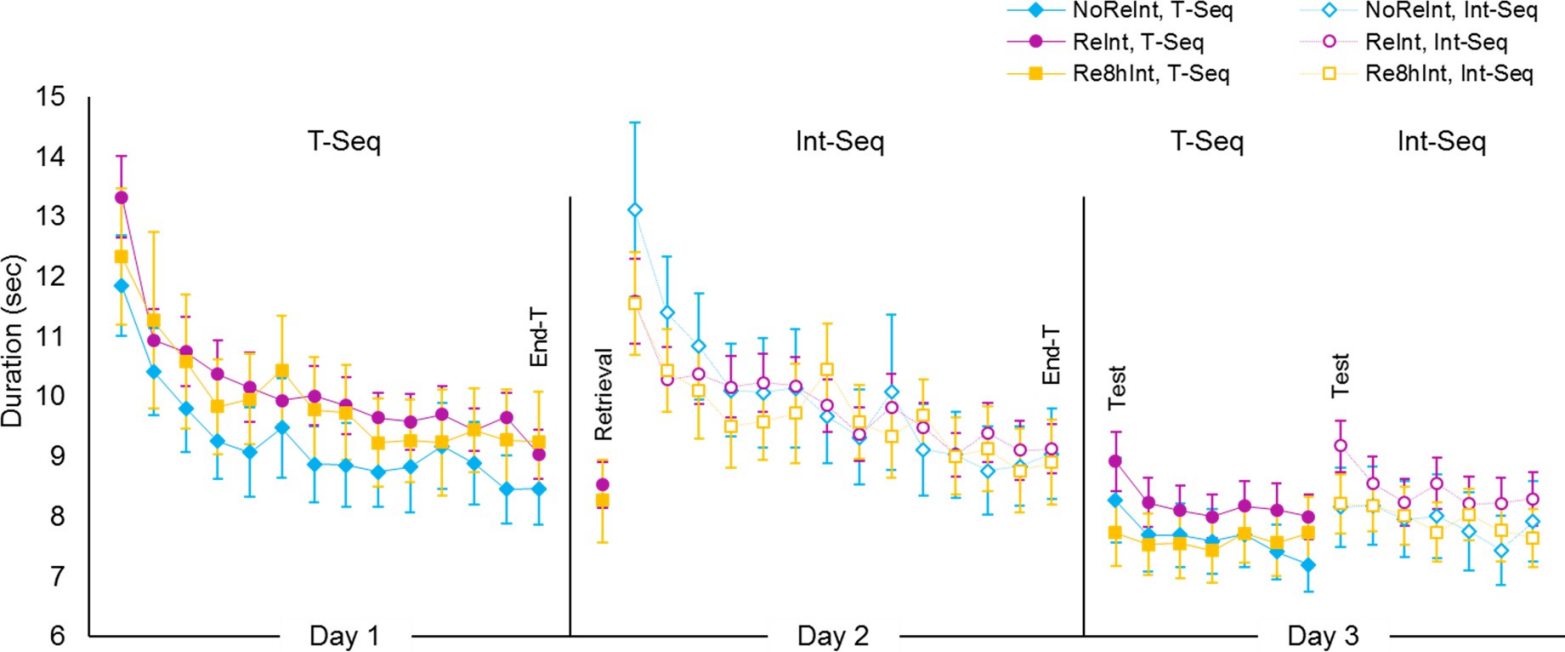
Performance rate. Time (i.e., duration) to complete the last 30 key-presses for each performance block during the training (Day 1), retrieval (Day 2) and test (Day 3) of the trained sequence (T-Seq, filled markers) as well as during the training (Day 2) and test (Day 3) of the interference sequence (Int-Seq, empty markers) is plotted for the NoReInt, ReInt and Re8hInt group (blue, magenta and yellow markers, respectively). Last training blocks, the retrieval block and first test blocks are time-points of interest (End-T, Retrieval and Test respectively). Bars–standard error of the mean (s.e.m.).

Throughout both training sessions, time on task for a single block averaged across groups shortened from 12.50 ± .50 sec during the first block to 8.92 ± .35 sec during the last block for the T-Seq, and from 12.08 ± .59 sec to 9.02 ± .35 sec for the Int-Seq (mean ± s.e.m.) (Fig 2, Day 1 and Day 2 respectively). These robust within-session improvements in performance rate were significant across sequences and groups, as indicated by a significant effect of *block* (*F*_(5.09, 239.41)_ = 40.01, *p* < .001) but no significant effect of *sequence* nor *group* (*F*_(1, 47)_ = .02, *p* = .89; *F*_(2, 47)_ = .11, *p* < .90 respectively). Although there was a trend towards a significant *sequence* by *group* interaction (*F*_(2, 27)_ = 3.02, *p* = .06), post hoc analyses performed separately for each *sequence* showed no significant effect of *group* (*F*_(2, 27)_ = .54, *p* = .59; *F*_(2, 47)_ = .05, *p* = .96, T-Seq and Int-Seq respectively). Comparison between the *sequences* within each *group* also failed to show significant difference (*F*_(1, 19)_ = 2.42, *p* = .13; *F*_(1, 19)_ = 2.45, *p* = .14 and *F*_(1, 19)_ = .72, *p* = .41, NoReInt, ReInt and Re8hInt group, respectively).

#### Tapping pattern of a sequence

**Fig 3:**
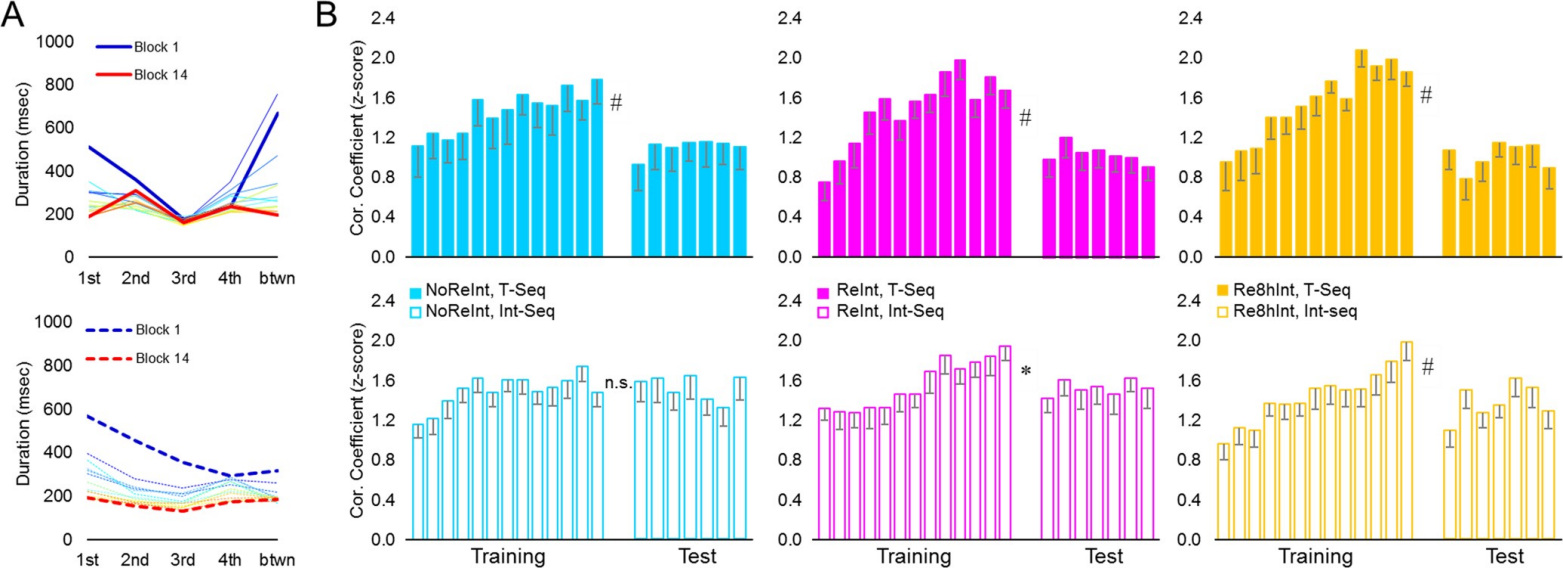
Experience-driven changes in tapping patterns. (A) Changes in tapping pattern, i.e., pattern of inter-key press intervals, from the first to the last block during the initial training on Day 1 and interference training on Day 2 (upper and lower plot respectively) are shown for one representative subject. Each point represents mean duration for each of 4 possible transitions between successive elements within a sequence (from 1st to 4th) plus an additional transition between the sequences (between) for each block. Thus, shape of each line depicts a tapping pattern for a single block. With practice, tapping pattern progressively became more similar to the tapping pattern generated during the last training block (red line). Note that changes in tapping pattern (i.e., line shape) do not directly reflect changes in performance rate. (B) Degree of similarity between tapping patterns was assessed based on normalized Pearson correlation coefficients using the Fisher’s z-transformation. Group average of individual normalized Pearson correlation coefficients between tapping patterns formed by the end of training (i.e., during the last training block) and each block during the training (training blocks 1–13) and test (test blocks 1–7) are shown for each sequence (T-Seq, upper plots; Int-Seq, lower plots). Bars–standard error of the mean (s.e.m.). *–significant results at .05 level, #–significant results at .01 level, n.s.–no significant differences.

During training, the initial tapping pattern of a sequence (Fig 3A) changed so that the degree of similarity to the tapping pattern formed by the end of training progressively increased from .94 ± .15 to 1.77 ± .11 for the T-Seq, and from 1.15 ± .08 to 1.80 ± .09 for the Int-Seq (mean ± s.e.m., correlation coefficients for the first and the penultimate training blocks, respectively) (Fig 3B, Training). These changes were highly significant and comparable across sequences and groups, as indicated by a significant effect of *block* (*F*_(6.94, 326.03)_ = 20.91, *p* < .001) with no significant effect of *sequence*, *group* nor any interaction (*F* < 1.24, *p* > .25). Thus, fast and robust gains in performance rate during the training were also associated with formation of a new, presumably more efficient kinematic pattern to generate each sequence. Our results indicate that motor skill levels achieved by the end of the initial training on Day 1 were comparable across groups. Moreover, memory encoding of a novel motor sequence knowledge on Day 2 was not affected by experience with the T-Seq on the previous day, nor by its retrieval.

### The effect of memory retrieval on the strength of the practiced motor skill

Next, we addressed our main research question by comparing performance levels expressed on Day 3 to those achieved by the end of training. This comparison allowed us to test the effect of memory retrieval on post-training gains in performance for each sequence.

#### Performance rate

**Fig 4:**
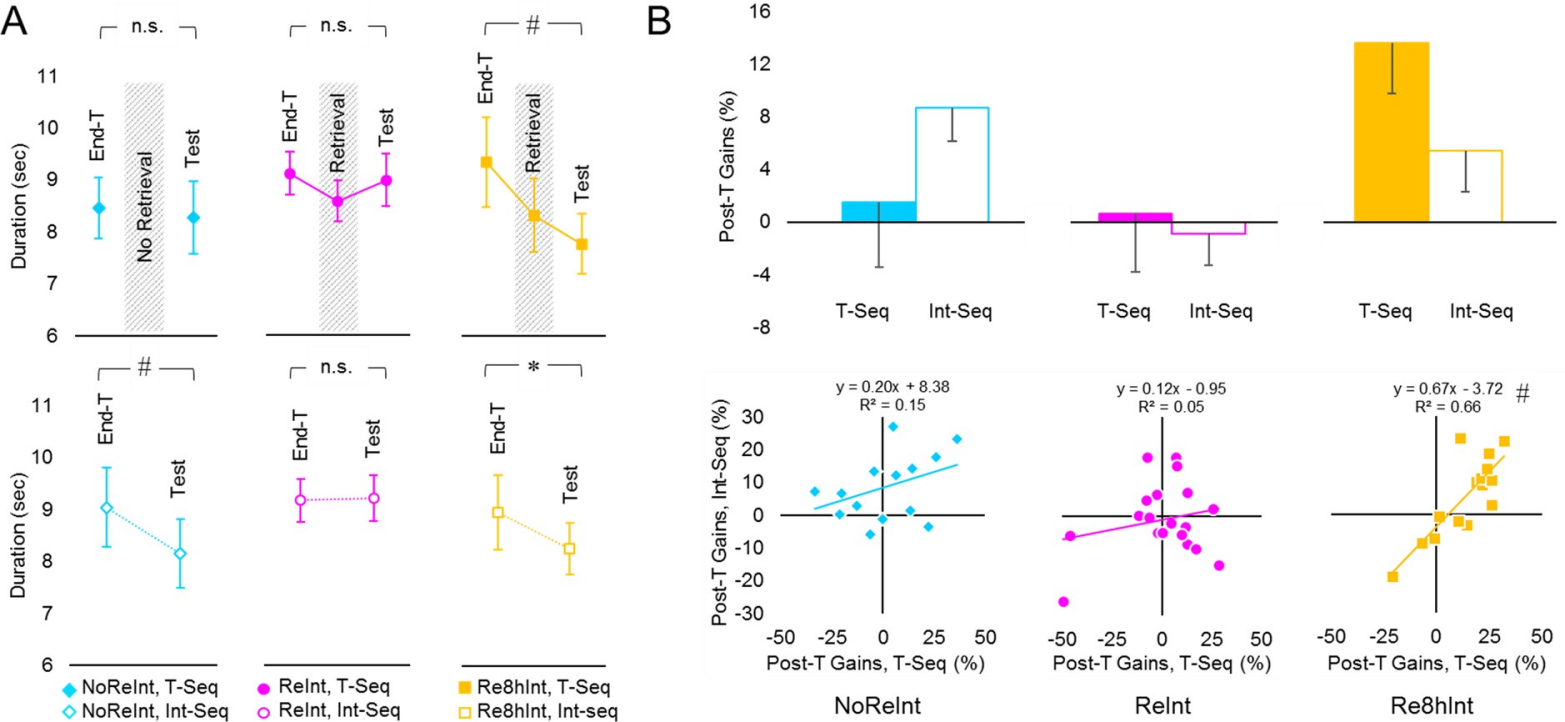
The effect of memory retrieval on performance rate. (A) Time to complete the last 30 key-presses during the last training block, the retrieval block and the first test block (End-T, Retrieval and Test respectively) is plotted for each group (NoReInt, ReInt and Re8hInt with blue, magenta and yellow markers respectively) and each sequence (T-Seq, filled markers upper plot; Int-Seq, empty markers lower plot). (B) Gains in performance, normalized to the last training block, that developed during the post-training interval for each sequence (i.e., between the end of training on Day 1 (T-Seq) or on Day 2 (Int-Seq) and the corresponding test session on Day 3; Post-T Gains) averaged across participants of each group (upper plot). Positive values correspond to faster performance by Day 3 than by the end of training. Individual Post-T Gains for the T-Seq (x axis) plotted against Post-T Gains for the Int-Seq (y axis) (lower plots). Bars–standard error of the mean (s.e.m.). *–significant results at .05 level, #–significant results at .01 level, n.s.–no significant differences. Note that in the ReInt group, regression analysis did not result in significant correlation even after excluding two participants with extremely negative values.

Analysis of performance duration resulted in significant effect of *time-point* (*F*_(1, 47)_ = 11.46, *p* = .001) with no significant effect of *sequence*, nor of *group* (*F*_(1, 47)_ = 1.00, *p* = .32; *F*_(2, 47)_ = .40, *p* = .67). There were, however, significant *time-point* by *group* and *time-point* by *sequence* by *group* interactions (*F*_(2, 47)_ = 3.58, *p* < .05; *F*_(2, 47)_ = 3.59, *p* < .05), suggesting substantial impact of retrieval processes on these post-training performance gains. To gain insight into differential effects of retrieval on off-line processes of each sequence, follow-up analyses were conducted separately for each sequence and each group (Fig 4A).

In the context of our main research question, remarkable results were observed in the NoReInt group that, similar to the ReInt group, failed to express additional post-training gains in performance rate for the T-Seq during the first test block on Day 3 (p = .67 and *p* = .75, NoReInt and ReInt group respectively) (Fig 4A, upper panel). Thus, by Day 3, the deficit in post-training gains for the T-Seq was observed regardless of whether or not memory for this sequence was retrieved immediately before the interference training. Note that significant post-training gains in performance rate for the T-Seq observed in the Re8hInt group (p < .01) suggest that short experience with the T-Seq during the retrieval block 8 hours prior to the interference training protected the retrieved memory from interference [25].

Comparing between performance levels achieved by the end of the interference training on Day 2 and during the corresponding test on Day 3, a deficit in post-training gains for the Int-Seq was observed only in the ReInt group (Fig 4A, lower panel). Without memory retrieval, however, participants in the NoReInt group developed robust gains during the post-training interval and performed the Int-Seq significantly faster on Day 3 than by the end of training on Day 2 (*p* < .01). The 8-hour interval between memory retrieval and interference training (Re8hInt group) also allowed for faster performance of the Int-Seq overnight (p < .05). Thus, memory retrieval juxtaposed to interference training prevented expression of post-training gains in performance not only for the T-Seq, but also for the Int-Seq. Note that given that the test for the Int-Seq was preceded by the test of the T-Seq, the possibility of proactive interference on the Int-Seq caused by additional experience with the T-Seq cannot be ruled out.

To test for potential effect of memory retrieval on the relationship between post-training gains expressed for each sequence on Day 3, regression analyses was conducted. To minimize the impact of individual differences in performance rate, post-training gains for each sequence were calculated in percentages relative to performance duration during the last training block so that positive values indicate additional improvement in performance, and negative values indicate performance deterioration (Fig 4B). To assess a moderation effect of memory retrieval, these values were mean-centered and entered into a hierarchical multiple regression analyses. In the first model, post-training gains for the T-Seq together with *group* factor (transformed to dichotomous variables) accounted for 28.5% of variance in consolidation gains of the Int-Seq (*r*^*2*^ = .285, *F*_(3, 46)_ = 6.10, *p* = .001). In the second model, the interaction term between post-training gains for the T-Seq and *group* significantly increased the explained variance by 10.6% (*r*^*2*^ change = .106, *F*_(2, 44)_ = 3.84, *p* < .05). The interaction between post-training gains for the T-Seq and *group* was also significant (*F*_(5, 44)_ = 5.65, *p* < .001), suggesting that the relationship between post-training gains for each sequence depended on memory retrieval. A significant correlation between the post-training gains for the T-Seq and the post-training gains for the Int-Seq was observed only for the Re8hInt group (*r* = .22, *p* = .35; *r* = .38, *p* = .16; *r* = .81, *p* < .001, ReInt, NoReInt and Re8hInt group respectively). Note that the results in the ReInt group failed to be significant even after excluding two participants with extremely negative values (*r* = .41, *p* = .09). Thus, only participants with an 8-hour delay between memory retrieval and interference training were able to express consolidation gains for the Int-Seq proportional to the total post-training gains for the T-Seq. This result suggests that active task-based memory retrieval followed by a “protective” 8-hour interval not only contributes to the strength of the retrieved motor memory but is also proportionally beneficial for consolidation of a new motor sequence knowledge. Upon other interference conditions, however, the lack of significant correlation between post-training gains of the two sequences rules out the possibility of direct trade-off between the impairment of the pre-established motor memory and consolidation of subsequently acquired new motor knowledge.

#### Tapping pattern of a sequence

By Day 3, the tapping pattern observed during the last training block underwent additional modification (Fig 3B). Analyses of the correlation coefficients of tapping pattern formed by the end of training (i.e., the last training block) with tapping pattern observed during each *time-point* of interest (the penultimate training block, the first test block) for each *sequence* (T-Seq, Int-Seq) resulted in a significant effect of *time-point* and *sequence* (*F*_(1, 47)_ = 61.35, *p* < .001; *F*_(1, 47)_ = 5.04, *p* < .05, respectively), as well as a significant *time-point* by *sequence* and a *time-point* by *sequence* by *group* interaction (*F*_(1, 47)_ = 5.49, *p* < .05; *F*_(2, 47)_ = 3.33, *p* < .05, respectively). These results suggest that during the post-training interval, tapping pattern formed by the end of training underwent significant changes that differed between sequences and groups. Post hoc comparisons performed separately for each *time-point* revealed that by the end of training all groups showed comparable degree of similarity between tapping patterns for both sequences (*F*_(1, 47)_ = .06, *p* = .81; *F*_(2, 47)_ = .96, *p* = .39; *F*_(2, 47)_ = 2.14, *p* = .13, effect of *sequence*, *group* and *sequence* by *group* interaction respectively). By Day 3, however, the degree of similarity of tapping pattern to the one formed by the end of training for the T-Seq was significantly lower than for the Int-Seq (*F*_(1, 47)_ = 10.14, *p* < .01) with no significant effect of *group* neither *sequence* by *group* interaction (*F*_(2, 47)_ = .31, *p* = .73; *F*_(2, 47)_ = 2.15, *p* = .13, respectively). Thus, during the post-training interval, tapping pattern for the T-Seq underwent greater modification compared to changes observed in the tapping pattern for the Int-Seq. These differences between the sequences did not depend on memory retrieval and may indicate that any experience with the task modifies its representation.

### Genuine memory impairment rather than retrieval deficit

Does the deficit in post-training performance gains reflect a genuine memory impairment as opposed to the initial difficulty in memory retrieval? We addressed this question in our last analyses by assessing changes in performance rate and dynamics in the tapping pattern of the sequence during the test session on Day 3.

#### Performance rate

Analyses of performance duration during the test of the T-Seq showed significant main effect of *block* across all groups (*F*_(4.36, 205.06)_ = 3.95, *p* < .01) (Fig 2, Day 3). The post-hoc analyses performed separately for each group, confirmed that the main effect of *block* was significant in the NoReInt and ReInt group (*F*_(6, 84)_ = 2.34, *p* < .05 and *F*_(3.52, 66.79)_ = 3.33, *p* < .05, respectively). These results indicate that the deficit in post-training performance gains for the T-Seq in these groups was associated with fast within-test improvement in performance rate. This improvement raises the possibility of a transient failure in motor memory retrieval rather than its genuine impairment [31]. Analyses of performance duration during the test of the Int-Seq showed similar results (main effect of *block*: *F*_(6, 84)_ = 2.24, *p* < .05 and *F*_(4.15, 78.83)_ = 4.28, *p* < .01, NoReInt and ReInt group respectively).

#### Tapping pattern of a sequence

**Fig 5:**
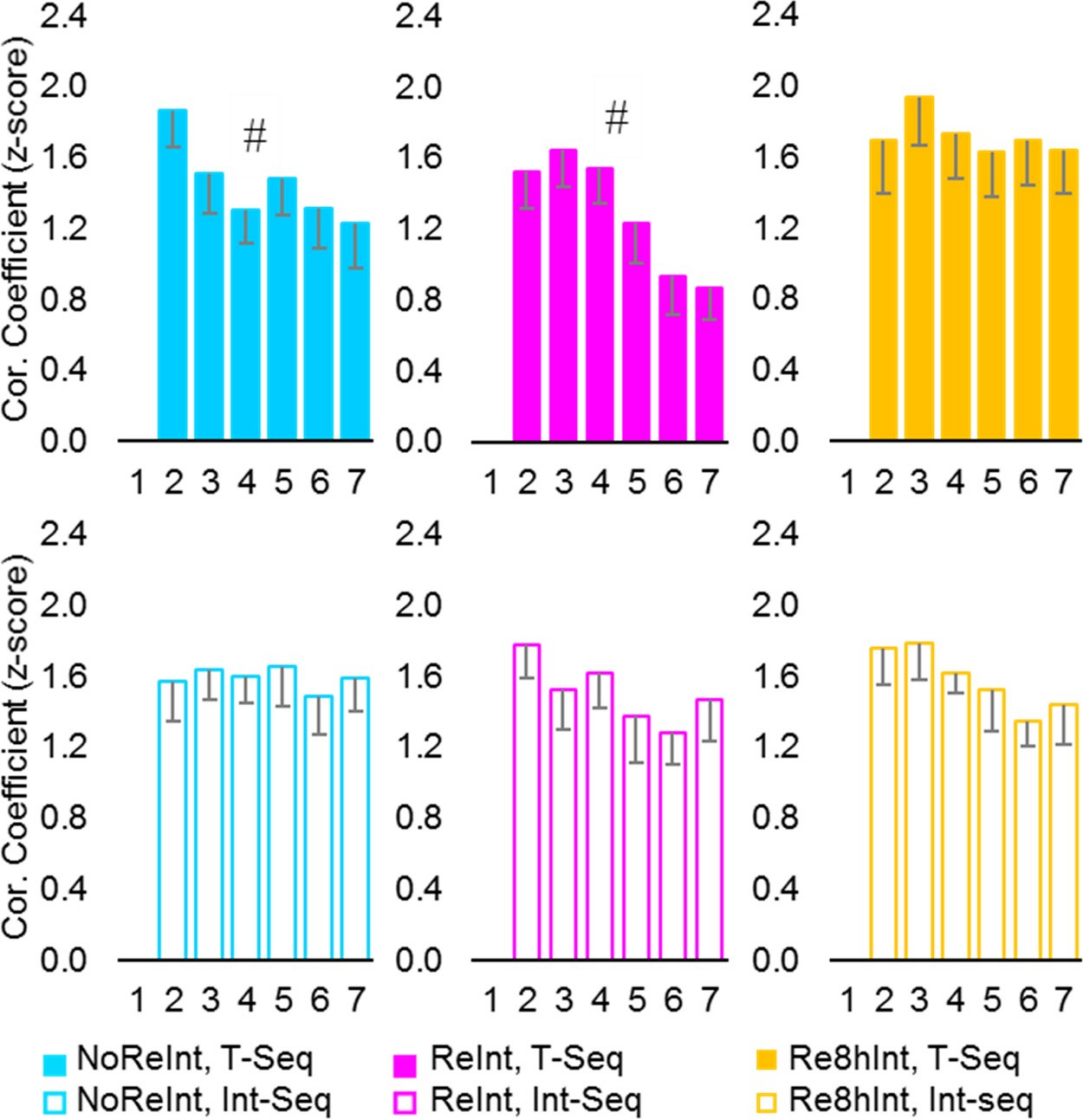
Changes in tapping patterns during test. Degree of similarity between tapping patterns, i.e., patterns of inter-key press intervals, was assessed based on normalized Pearson correlation coefficients calculated for each individual using the Fisher’s z-transformation. Mean normalized Pearson correlation coefficients between tapping patterns for the first test block and during the subsequent repeated practice (i.e., test blocks 2–7) for each group and each sequence (T-Seq, upper plots; Int-Seq, lower plots). Bars–standard error of the mean (s.e.m.). #–significant results at .01 level.

Based on our previous results [25], we assumed that if within-test improvement in performance rate reflects an initial failure in memory retrieval and its rapid recovery with continued practice, then we should also find evidence for recovery of the previously formed tapping pattern. However, the analyses of the T-Seq tapping pattern dynamics during the test blocks, versus the last training block on Day 1, showed no significant effect of *block* (*F*_(6, 84)_ = .52, *p* = .79; *F*_(2.64, 50,14)_ = .59, *p* = .61 and *F*_(3.60, 50,40)_ = 2.30, *p* = .08, NoReInt, ReInt and Re8hInt group respectively) (Fig 3B, upper plots), providing no evidence for recovery of the previously formed tapping pattern. Therefore, retrieval failure as a possible explanation for performance deficit for the T-Seq on Day 3 observed in the NoReInt group, similar to the ReInt group, is not supported. Yet, in both these groups, fast within-test improvement in performance rate of the T-Seq was paralleled by significant within-test changes in its tapping pattern. These changes were evident while assessing the degree of tapping patterns’ similarity between the first and the remaining (i.e., 6 out of the 7) test blocks (main effect of *block*: *F*_(3.88, 182.26)_ = 6.31, *p* > .001; *block* by *group* interaction: *F*_(7.76, 182.26)_ = 2.02, *p* > .05; post-hoc analyses showed main effect of *block* for the NoReInt and ReInt group: *F*_(5, 70)_ = 3.92, *p* > .01 and F_(5, 95)_ = 8.37, p < 0.001, respectively) (Fig 5, upper plots). Thus, the rapid within-test improvement in performance rate of the T-Seq observed in the NoReInt group, similar to the ReInt group, did not result from a simple performance acceleration or delayed memory retrieval, but rather required or occurred due to a generation of a new, presumably more efficient, tapping pattern. In the Re8hInt group, on the other hand, no significant within-test changes in the tapping pattern of the T-Seq were observed, indicating reliable and stable task representations.

Assessing tapping pattern dynamics during the test session of the Int-Seq, noticeable results were observed only in the Re8hInt group. Across test blocks, the degree of tapping pattern similarity to the one formed by the end of interference training increased (*F*_(3.12, 43.69)_ = 3.11, *p* > .05) (Fig 3B, lower right plot). This recovery of previously formed tapping pattern for the Int-Seq in the Re8hInt group was also associated with a trend towards decreased tapping pattern similarity to the one observed during the initial test block (*F*_(5, 70)_ = 2.25, *p* = .06) (Fig 5, lower right plot).

## Discussion

The main goal of our study was to examine the necessity of memory retrieval to induce a deficit in a pre-established procedural (“how to”) motor memory in humans [17]. To this end, we used a 3-day motor sequence learning paradigm which consisted of an initial training on Day 1, an interference training on Day 2, preceded or not by the actual task-based retrieval of the initially trained sequence, and a final test session on Day 3. Our results suggest that susceptibility of consolidated motor memory to behavioral interference is independent of its active task-based retrieval. Since active task-based retrieval is the most adequate mechanism to reactivate memories [49], the impairment of the pre-established motor skill in the absence of its retrieval challenges the reconsolidation view that considers memory reactivation as a necessary condition for susceptibility of consolidated memory to changes [5,6,9,19].

Yet, one may argue that upon exposure to the same context where the initial training was given, the memory trace for the trained sequence in the NoReInt group could have been spontaneously reactivated and temporarily destabilized even without actual / physical memory expression [21–24]. Indeed, human studies suggest that declarative and fear memories can also undergo reconsolidation-like processes following the presentation of the learned cue only [11,15,50]. If the re-exposure to the experimental environment may lead to spontaneous motor memory trace reactivation, then participants in the Re8hInt group should have also shown impairment of the pre-established motor skill. Participants in this group left the laboratory after the retrieval experience. They were instructed to continue with their regular routine and were re-exposed to the experimental environment during the interference training 8 hours later. However, on Day 3 the post-training gains in the Re8hInt group were comparable to the post-training gains expressed by participants that on Day 2 underwent memory retrieval without subsequent interference training (i.e., ReactNoInt group in our previous report [25]). Consequently, the hypothetical possibility for spontaneous memory reactivation by experimental context and its subsequent destabilization contradicts the absence of interference effects in the Re8hInt group and, therefore, is unlikely.

We suggest that the current pattern of results supports a memory integration model [1,35]. This model refers to the idea that memories for related experiences are stored as overlapping representations in the brain. Previous experience with the task and memory trace dominance may dictate the degree of such overlap influencing the way new information is stored. Accordingly, rather than memory destabilization and recapitulation of initial consolidation processes, memory reactivation immediately before interference training may have facilitated integration of new knowledge into previously established memory trace through its consolidation. The high overlap between neuronal ensembles representing memories for two different sequences could lead to mutual interference between these sequences [30,51,52]. Indeed, in the ReInt group, memory retrieval juxtaposed to interference training prevented expression of post-training performance gains not only for the T-Seq, but also for the Int-Seq. The integration model can also explain the time-dependent effect of retrieval experience on these gains in the ReInt and Re8hInt group.

The detrimental effect of interference training on the pre-established motor memory in the NoReInt group, similar to the ReInt group, was expressed as a lack of additional post-training gains, so that the performance rate for the T-Seq on Day 3 did not significantly differ from its performance rate achieved by the end of the initial training on Day 1 (Fig 4A, upper panel). Post-training delayed gains in motor skill levels are considered to be a behavioral signature for successful motor sequence memory consolidation [9,26–28,53,54]. Consolidation processes require time and, following practice on an explicitly introduced motor sequence, time-in-sleep to become effective [27,42,55–57]. Indeed, when participants performed the T-Seq as a retrieval procedure on Day 2, their performance was significantly faster than by the end of the initial training on Day 1 [25]. Given the very same training experience on Day 1, one would have expected similar consolidation gains in the NoReInt group on Day 2. Therefore, we assume that the lack of additional post-training gains on Day 3 in the NoReInt group, similar to the ReInt group, indicates that the detrimental effect of interference training on the pre-established motor memory was expressed as a loss of consolidation gains.

If the loss of consolidation gains for the T-Seq is the consequence of a failure in restabilization of the pre-established motor sequence knowledge [10], one would also have expected a greater reliance on task representation formed by the end of the initial training, i.e., before the newly encoded memory was stabilized through consolidation. However, during the test session on Day 3, performance of the T-Seq was characterised by decreased reliance on tapping pattern formed by the end of the initial training on Day 1 in all groups (Fig 3B, upper plots). This pattern of result did not change even after additional experience with the T-Seq on Day 3, as indicated by analyses of tapping pattern dynamics across test blocks, providing no evidence for rescue of previously established tapping pattern with continuous practice. Thus, intact and impaired performance levels for the T-Seq, as indicated by expression of consolidation gains in the Re8hInt group and their abolition in the ReInt and NoReInt group, respectively, were associated with decreased reliance on initially established task representation. Therefore, restabilization failure as a possible explanation for the loss of consolidation gains following behavioral interference is not supported.

On Day 3, the loss of consolidation gains for the T-Seq was also associated with fast within-test improvement in performance rate. Did this improvement reflect an initial failure in memory retrieval and its rapid recovery with continuous practice? If so, the within-test improvement in performance rate should have been paralleled by greater reliance on the previously established tapping pattern. However, the decreased similarity between the tapping pattern of the T-Seq on Day 3 and the tapping pattern formed by the end of the initial training did not significantly changed throughout the test blocks (Fig 3B, upper plots), providing no evidence for memory recovery. Yet, the tapping pattern of the T-Seq in the NoReInt group, similar to the ReInt group, underwent significant changes so that the degree of similarity to the tapping pattern generated by each participant during the first test block progressively decreased with practice (Fig 5, upper plots). These characteristic changes in the tapping pattern observed during the test were analogous to those observed during the initial training [25]. We propose that the loss of consolidation gains for the T-Seq in the NoReInt group, similar to the ReInt group, implies a genuine impairment of the motor skill memory. The explanation of a transient deficit in memory retrieval is not supported.

Significant post-training gains for the T-Seq in the Re8hInt group, but not upon other interference conditions, suggest that short retrieval experience followed by the “protective” 8-hour interval was sufficient to prevent unfavorable effects of the interference training even upon a hypothetical possibility for spontaneous memory reactivation by experimental context *per se*. It has been recently shown that a brief memory retrieval can result in significant learning and improve visual perception [58]. Moreover, repetitive task-based retrieval of the pre-established motor memory interleaved with new learning can also enable prevention of interference for the long term [59]. In line with these findings, our results suggest that active task-based memory retrieval can significantly contribute to the memory strength [58] and protect it from future interference in a time-dependent manner.

Despite similar impairment in performance observed for the T-Seq in the NoReInt and ReInt group by Day 3, two ways of conjectural memory reactivation, with or without its actual retrieval, resulted in differential competition between overlapping memories, as indicated by consolidation gains for the Int-Seq in the NoReInt group, but not in the ReInt group (Fig 4). These results are in line with the idea that trace dominance, determined by the intensity of previous experience in general and memory reactivation in particular, plays a crucial role in synergistic and competitive interactions between memories [48,60–62]. Here, similar performance levels and comparable improvements paralleled by changes in a tapping pattern were observed during both initial and interference training sessions in all groups. Therefore, the differential expression of post-training consolidation gains for the Int-Seq cannot be explained by differences in initial memory encoding and is more likely related to conjectural reactivation of overlapping, but distinct neural populations elicited with and without actual retrieval experience (ReInt and NoReInt group, respectively). It is not clear, however, whether the lack of spontaneous consolidation gains for the Int-Seq in the ReInt group resulted from competition between overlapping engrams during consolidation phase or during the recall. The possibility of proactive interference caused by additional experience with the T-Seq prior to the test of the Int-Seq cannot be ruled out [40]. In any case, the 8-hour interval between memory retrieval and re-exposure to experimental context during interference training allowed participants of the Re8hInt group to express significant post-training gains for both sequences. The observation that only in the Re8hInt group individual consolidation gains for the Int-Seq were proportional to the total post-training gains for the T-Seq supports the idea of time-dependent overlap and separation between retrieved and new memories.

The engagement of overlapping neuronal populations to represent consolidated and new memories may be governed by similar transient competitive processes that link distinct new memories encoded close in time [63–65]. It has been shown that learning triggers a temporary increase in intrinsic excitability of encoding neurons [66]. This increase contributes to a stronger stimulation of a particular synaptic pathway that leads to two dissociable events: local tag setting and the synthesis of diffusible plasticity related proteins (PRPs) [65] increasing the probability of the tagged synapses to be involved in encoding of another task learned shortly after [64]. The persistence of encoded memory traces depends upon consolidation processes through synaptic capture of PRPs [63,65]. Since the availability of PRPs generated in the cell bodies is limited, during consolidation of two memories encoded close in time, a “winner-take-all” scenario appears to prevail whereby some encoded traces persist in a stable manner whereas others do not [60]. Thus, transient increases in intrinsic neuronal excitability and competitive synaptic tagging and capture mechanism determine the distribution of synapses that are strengthened or weakened during learning [62]. Recently, studies on animal models provided convincing support for the transient and competitive nature of these processes demonstrating greater overlap between memories encoded close in time and greater separation between memories encoded farther apart in time [67,68]. This time-dependent co-allocation of memory traces may not be limited to linking new memories and may also occur following memory reactivation experience given immediately before new learning, as in the ReInt group, but not with 8-hour delay. Activation of overlapping networks during memory retrieval may create an interference problem at a behavioral level [52]. Thus, engagement of overlapping populations of neurons during encoding and “winner-take-all” competition for resources during memory consolidation provide a plausible framework for interpreting impaired performance associated with both, consolidation failure induced by new learning shortly after initial training [55,56,69,70] and the so-called “restabilization” failure allowed through memory reactivation [9,10,14,15,71,72].

It is noteworthy that during the post-training interval, greater changes in tapping pattern for the T-Seq, versus the Int-Seq, were observed in all groups irrespective of the retrieval-interference procedure (Fig 3B). These differences could be explained by unequal time interval after the end of each training session. However, this explanation is not supported by our previous observations [25]. By Day 3, the group that had no experience with the task during the post-training interval showed greater reliance on tapping pattern formed by the end of the initial training on Day 1 compared to all other groups. Thus, rather than a simple passage of time, our results suggest that any experience with the task modifies its representation. These changes can either strengthen or weaken previously established memories resulting in impaired or improved performance.

In conclusion, here we showed that, in humans, active task-based memory retrieval of the pre-established motor skill is not necessary to induce its impairment through behavioral interference. However, even upon the hypothetical possibility for spontaneous memory reactivation by experimental context without its actual task-based retrieval, the post-reactivation failure of memory restabilization proposed by the reconsolidation theory is not supported. Instead, the current pattern of results supports a memory integration model according to which reactivation-induced brain plasticity influences the way new information is stored and may facilitate its integration into previously established memory trace through its consolidation.

## Supporting information

S1 Table Demographic information and sleep duration. (PDF)

S1 File Supplementary results. (PDF)
