# Supplementary material for: Susceptibility of consolidated procedural memory to interference is independent of its active task-based retrieval
Source: PLoS One. 2019 Jan 17;14(1):e0210876. doi: 10.1371/journal.pone.0210876 (PMC6336251; doi:10.1371/journal.pone.0210876)
Supplement: S1 Table — (PDF) [file pone.0210876.s001.pdf]

Demographic information and sleep duration.

Group	Females (N)	Males (N)	Age (years)	Night sleep 1 (self report)	Night sleep 2 (self report)
NoReInt	7	8	23.16 ± 2.38	7h 50 min ± 49 min	8h 2 min ± 53 min
ReInt	11	9	25.25 ± 4.56	8h 5 min ± 57 min	8h 2 min ± 53min
Re8hInt	10	5	24.60 ± 3.53	7h 34 min ± 49 min	7 h 24 min ± 56 min
