# Supplementary material for: Susceptibility of consolidated procedural memory to interference is independent of its active task-based retrieval
Source: PLoS One. 2019 Jan 17;14(1):e0210876. doi: 10.1371/journal.pone.0210876 (PMC6336251; doi:10.1371/journal.pone.0210876)
Supplement: S1 File — (PDF) [file pone.0210876.s002.pdf]

Supplementary results

Accuracy during encoding of a new motor sequence knowledge

Performance on both trained and interference sequences was characterized by high levels of accuracy (96.22 ± 0.25 % and 95.50 ± 0.28 %, mean ± s.e.m across training blocks for the T-Seq and Int-Seq respectively) that did not change significantly throughout the training sessions. Repeated-measures ANOVA with *sequence* (T-Seq, Int-Seq) and *block* (14 training blocks) as within-subject factors and *group* (ReNoInt, NoInt and Re8hInt) as a between-subject factor showed no significant effect of *sequence, block* nor *group* ($F_{(9.42, 442.64)} = 1.33$, $p = .21$; $F_{(1, 47)} = 1.96$, $p = .17$; $F_{(2, 47)} = .91$, $p = .41$, respectively) as well as no significant interactions ($F < 1.52$, $p > .14$). These results suggest that during training, performance of either sequence in all experimental groups was characterised by high levels of accuracy that did not change significantly with practice. Moreover, accuracy levels during training on a novel sequence on Day 2 were not affected by the preceding retrieval of the initially trained and consolidated sequence.

The effect of memory retrieval on accuracy

Analyses of the percentage of correct transitions did not yield significant effects with any of the factors ($F_{(1, 47)} = .26$, $p = .61$; $F_{(1, 47)} = .18$, $p = .68$; $F_{(2, 47)} = .96$, $p = .40$, *sequence*, *time-point* and *group* respectively) but showed a significant *sequence* by *group* interaction ($F_{(2, 47)} = 4.95$, $p = .01$). Although post hoc analyses showed that performance of the T-Seq, compared to the Int-Seq across time-points of interest, was significantly less accurate in the NoReInt group and more accurate in the ReInt group ($F_{(1, 14)} = 6.48$, $p < .05$ and $F_{(1, 19)} = 4.48$, $p < .05$ respectively), the effect size was very small (less than 2.6% for either group). Moreover, the absence of significant effect of *time-point* and its interaction with other factors ($F < 1.04$, $p > .36$) indicates that accuracy levels did not change significantly over the post-training interval regardless of previous experience with the task gained during the initial training on Day 1 or during the retrieval block on Day 2.

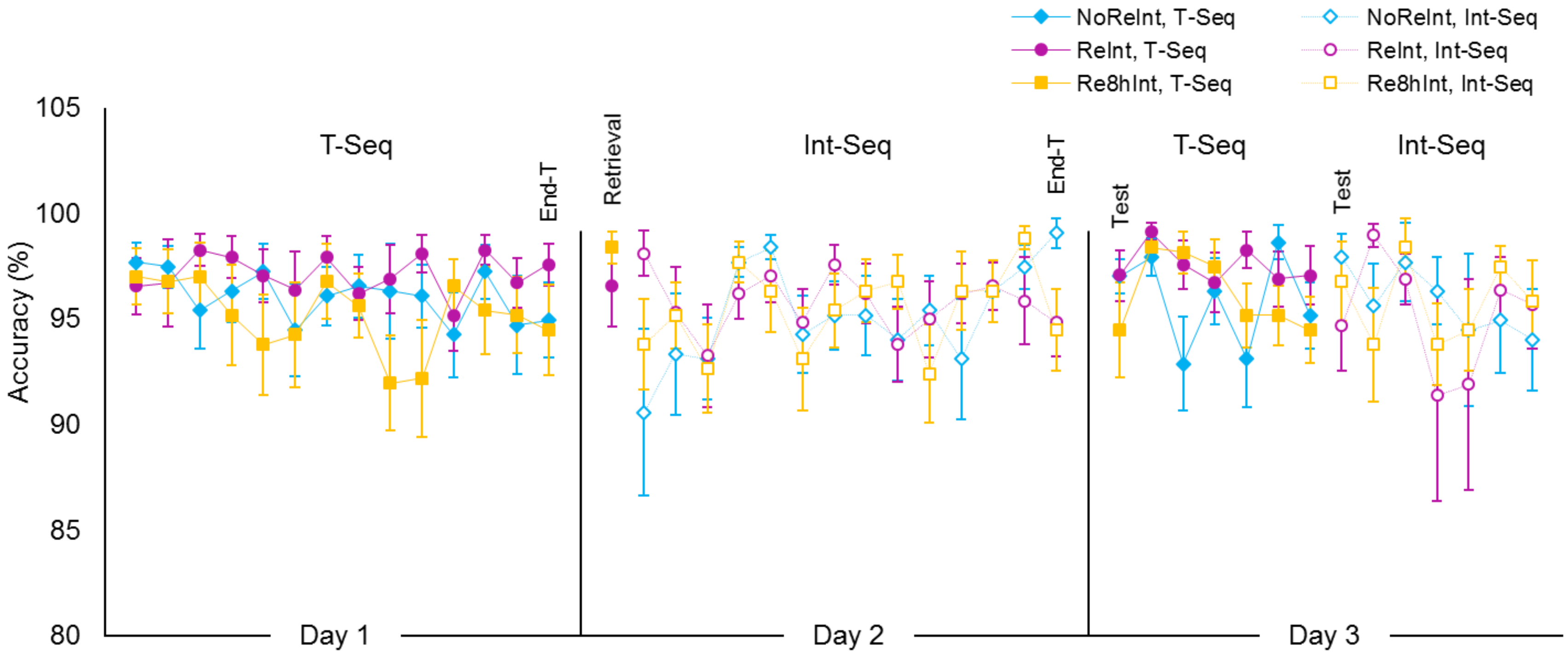

Accuracy. The percentage of correct key-presses (out of the last 30) for each performance block during the training (Day 1), retrieval (Day 2) and test (Day 3) of the trained sequence (T-Seq, filled markers) as well as during the training (Day 2) and test (Day 3) of the interference sequence (Int-Seq, empty markers) is plotted for the NoReInt, ReInt and Re8hInt group (blue, magenta and yellow markers, respectively). Last training blocks, the retrieval block and first test blocks are time-points of interest (End-T, Retrieval and Test respectively). Bars – standard error of the mean (s.e.m.).
